# Impact of Stock Market Structure on Intertrade Time and Price Dynamics

**DOI:** 10.1371/journal.pone.0092885

**Published:** 2014-04-03

**Authors:** Plamen Ch. Ivanov, Ainslie Yuen, Pandelis Perakakis

**Affiliations:** 1 Center for Polymer Studies and Department of Physics, Boston University, Boston, Massachusetts, United States of America; 2 Harvard Medical School and Division of Sleep Medicine, Brigham and Women's Hospital, Boston, Massachusetts, United States of America; 3 Institute of Solid State Physics, Bulgarian Academy of Sciences, Sofia, Bulgaria; 4 Signal Processing Laboratory, Department of Engineering, Cambridge University, Cambridge, United Kingdom; 5 Laboratory of Experimental Economics, University Jaume I, Castellón, Spain; 6 Mind, Brain and Behaviour Research Centre (CIMCYC), University of Granada, Granada, Spain; University of Maribor, Slovenia

## Abstract

We analyse times between consecutive transactions for a diverse group of stocks registered on the NYSE and NASDAQ markets, and we relate the dynamical properties of the intertrade times with those of the corresponding price fluctuations. We report that market structure strongly impacts the scale-invariant temporal organisation in the transaction timing of stocks, which we have observed to have long-range power-law correlations. Specifically, we find that, compared to NYSE stocks, stocks registered on the NASDAQ exhibit significantly stronger correlations in their transaction timing on scales within a trading day. Further, we find that companies that transfer from the NASDAQ to the NYSE show a reduction in the correlation strength of transaction timing on scales within a trading day, indicating influences of market structure. We also report a persistent decrease in correlation strength of intertrade times with increasing average intertrade time and with corresponding decrease in companies' market capitalization–a trend which is less pronounced for NASDAQ stocks. Surprisingly, we observe that stronger power-law correlations in intertrade times are coupled with stronger power-law correlations in absolute price returns and higher price volatility, suggesting a strong link between the dynamical properties of intertrade times and the corresponding price fluctuations over a broad range of time scales. Comparing the NYSE and NASDAQ markets, we demonstrate that the stronger correlations we find in intertrade times for NASDAQ stocks are associated with stronger correlations in absolute price returns and with higher volatility, suggesting that market structure may affect price behavior through information contained in transaction timing. These findings do not support the hypothesis of universal scaling behavior in stock dynamics that is independent of company characteristics and stock market structure. Further, our results have implications for utilising transaction timing patterns in price prediction and risk management optimization on different stock markets.

## Introduction

The impact of market structure and associated rules of operation on market efficiency and stock price formation have attracted considerable public attention [1]. Developments on the New York Stock Exchange (NYSE) [1], [2], have raised the profile of the market operating mechanism, the “market structure”, employed by a stock market. This has also been of concern to those involved in stock market regulation, on behalf of investors [1], [3], since optimizing market structure results in more effectively functioning markets [4] and increases competitiveness for market share in listed stocks [5]. The two major stock markets in the U.S., the NYSE and the National Association of Securities Dealers Automated Quotation System (NASDAQ) National Market have very different structures [6], [7], and there is continuing controversy over whether reported differences in stock price behavior are due to differences in market structure or company characteristics [8]. Comparative studies of the NYSE and NASDAQ have primarily focused on stock prices to provide evidence that market organizational structure affects the price formation process [4], [9], [10]. It has been shown that stocks registered on the NASDAQ may be characterized by a larger bid-ask spread [11] and higher price volatility [4], [9], [10]. However, this is often attributed to the market capitalization, growth rate or the nature of the companies listed on the NASDAQ [8]. Empirical studies have also emphasized the dominant role and impact of trading volume on prices [12], [13]; since traded volume is determined by investors it is difficult to isolate the effects of market structure on price formation. As the influence of market structure on stock prices may be obscured by exogenous factors such as demand and supply [12], [13], we hypothesize that modulation of the flow of transactions due to market operations may carry a stronger imprint of the internal market mechanism.

Here we analyse times between consecutive transactions for a diverse group of stocks registered on the NYSE and NASDAQ markets, and we relate the dynamical properties of the intertrade times with those of the corresponding price fluctuations. To understand how market structure may affect stock prices, we study the information contained in the times between consecutive stock transactions. As market-specific operations may modulate the flow of transactions, we hypothesize that dynamical features of transaction timing reflect the underlying market mechanism. Specifically, we ask if stocks of companies with diverse characteristics registered on a given market exhibit common features in their transaction timing, which may be associated with the particular market structure. Further, we investigate how the dynamical properties of transaction timing over a range of time scales relate to stock price dynamics and whether market structure affects the temporal organisation of price fluctuations.

To probe how market structure influences the trading of stocks, we consider the two major U.S. stock markets, the NYSE and the NASDAQ. All transactions on the NYSE of a given stock are centralised and are controlled by a *single* human operator called a “specialist”, whose primary role is to match together public buy and sell orders on the basis of price, in an auction-like setting [6]. The NYSE specialist is under obligation to maintain both price continuity and a “fair and orderly market” [6], as well as to intervene, using his own firm's inventory of available stock, to provide liquidity in the event of an order imbalance, thus preventing sharp changes in the stock price [6]. The NYSE regulations allow for considerable flexibility within the specialist's operations [2].

In contrast, trading on the NASDAQ is decentralised, with trading in a given stock managed by a *number* of dealers called “market makers”. These market makers maintain a stock inventory, posting their best prices at which they are prepared to immediately buy and sell stock [7]. Market makers compete with each other for orders, so in theory competition ensures that investors get the best prices. Alternatively, an order can be placed into an Alternative Trading System (ATS), operated by NASD members or NASD-member affiliates and designed to allow two subscribers to meet directly on the system under the regulation of a third party. The most commonly used form of ATS is the Electronic Communication Network (ECN), a facility that matches customer buy and sell orders directly through a computer network.

A third alternative, in case the order placed is very small, is to enter the order into the Small Order Execution System (SOES), which is an electronic network designed to allow fast automatic routing, execution and reporting of orders of 500 shares or less. Orders are automatically routed to market makers whose quotes are currently identical to the highest bid (buy) and the lowest offer (sell) prices. Participation in the SOES system was made mandatory [7] after the market crash of October 1987, as one of the reported problems on the NASDAQ during the crash was the inability to reach market makers by the phone during periods of rapid price movement.

To summarize the differences between the two market structures, each market maker on the NASDAQ maintains his own inventory of stock in order to buy and sell [7]. In comparison, the NYSE specialist rarely uses his own firm's inventory: such transactions involve less than 15% of trading volume [14]. Although several regional exchanges may trade NYSE listed stocks, price formation has primarily been attributed to NYSE trading [15]. In contrast, the NASDAQ market relies on competition between multiple dealers for public orders to facilitate the price formation process [11]. Moreover, a substantial fraction of share volume on the NASDAQ is not handled by dealers, but is traded electronically via networks for small public orders and for institutional investors [7]. Such fragmentation of the NASDAQ stock market has been associated with higher price volatility [4].

**Figure 1:**
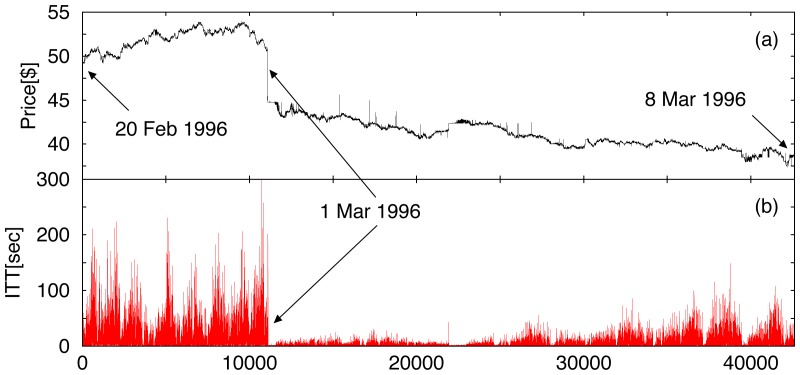
Relationship between stock price and trading activity. Representative example of time series derived from the Trades and Quotes (TAQ) database for transactions of stock in Compaq Computer Corp. (CPQ) registered on the NYSE. (a) Price of CPQ stock over a three week period from 20 Feb.- 8 Mar. 1996 (42606 trades). On 1 Mar. 1996 Compaq reported that it would cut product prices in order to meet sales targets, leading to a drop in the stock price. (b) Intertrade times (ITT) of CPQ stock over the same period. Data exhibit complex fluctuations, a daily pattern of trading activity (with short ITT at the open and close of a trading day and longer ITT in between), and highly heterogeneous structure, as seen in the flurry of trades following the price drop. The relaxation time of the ITT response following the price drop extends over several days, suggesting that information may be contained in the temporal structure of trading activity. Data include transactions occurring between 9.30am and 4pm EST, excluding weekends and holidays.

Here we ask to what extent such structural and operational differences between the NYSE and NASDAQ markets affect the flow of transactions. It is difficult to answer whether differences in intertrade times are due to individual company characteristics or external market influences (Fig. 1). Two empirical studies have considered only a single company stock over a short period of a few months [16], [17]. Studies which considered a larger group of stocks either did not find common features in the intertrade times [18], [19] or did not compare between markets [20]–[22]. The only comparative study considered a single NYSE and a single Paris stock, finding some differences in their intertrade times, but those may well be due to a different culture of trading [23]. To probe for evidence of the impact of market structure on the trading of stocks, we employ concepts and methods from statistical physics to investigate the correlation properties of transaction timing for diverse companies, over time scales ranging from seconds up to a year.

## Data

We examine one hundred stocks listed on the NYSE, from eleven industry sectors: Technology-Hardware(5), Semiconductors(2), Pharmaceutical & Medical Equipment(10), Financial(8), Automotive(9), Defense/Aerospace(9), Mining, Metals & Steel Works(8), Chemicals & Plastics(7), Retail & Food(17), Petroleum, Gas & Heavy Machinery(10), Telephone Service Providers(7), Electric & Power Services(8). We study the time intervals between successive stock trades, over a period of four years–4 Jan. 1993 to 31 Dec. 1996–as recorded in the Trades and Quotes (TAQ) database from the NYSE (Table 1).

**Table 1:** Characteristics of one hundred NYSE stocks studied over the period 4 Jan. 1993–31 Dec. 1996.

Company Name(Ticker Symbol)	Industry	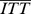 Numberof Trades	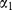 (sec)	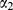 ITT	ITT	Company Name(Ticker Symbol)	Industry	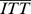 Numberof Trades	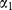 (sec)	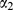 ITT	ITT
Meredith (MDP)	Food & Retail	35267	636	0.6	0.75	Medtronics (MDT)	Medical Apparatus	308049	75	0.6	0.88
Transco (E)	Natural Gas	47045	405	0.62	0.79	Southern (SO)	Electric Services	329464	71	0.64	0.85
Avery Dennison (AVY)	Paper Products	62927	365	0.59	0.75	Schlumberger (SLB)	Oil & Gas	330830	70	0.61	0.78
Johnson Controls (JCI)	Automatic Controls	68490	334	0.56	0.73	Amoco (AN)	Petroleum	339996	69	0.6	0.75
Northrop Grumman (NOC)	Aerospace/Defense	69739	330	0.6	0.81	PG & E (PCG)	Electric Services	355190	66	0.64	1.02
Allergan (AGN)	Pharmaceutical	71419	322	0.6	0.8	Sprint PCS (FON)	Telephone Comms.	362851	64	0.63	0.92
Jefferson Pilot (JP)	Financial	79013	292	0.58	0.77	Homestake Mining (HM)	Mining	370132	63	0.7	0.89
Nalco Chemical (NLC)	Chemicals	81731	283	0.58	0.72	Union Carbide (UK)	Chemicals	387273	60	0.64	0.96
Lockheed Martin (LK)	Aerospace/Defense	44897	282	0.58	0.74	Nynex (NYN)	Telephone Comms.	386703	60	0.61	0.87
Northern States Pow. (NSP)	Electric Services	85724	269	0.59	0.76	Morgan J.P. & Co. (JPM)	Financial	401213	58	0.61	0.87
Dana (DCN)	Automotive	89700	257	0.59	0.75	Dow Chemical (DOW)	Chemicals	411258	57	0.62	0.94
Inland Steel Ind. (IAD)	Steelworks	91137	253	0.6	0.88	Mobil (MOB)	Petroleum Refining	430401	54	0.62	0.76
Ashland Inc. (ASH)	Petroleum Refining	94396	245	0.59	0.77	Schering Plough (SGP)	Pharmaceutical	431388	54	0.62	0.84
General Dynamics (GD)	Aerospace/Defense	97594	237	0.58	0.83	Chase Manhattan (CMB)	Financial	448801	52	0.65	0.94
Eaton (ETN)	Automotive	98796	234	0.58	0.76	BellSouth (BLS)	Telephone Comms.	450144	52	0.63	0.86
Ethyl (EY)	Chemicals	100663	229	0.61	0.84	3M (MMM)	Paper Products	449462	52	0.61	0.83
TRW Inc. (TRW)	Automotive	111506	208	0.58	0.81	Texaco (TX)	Petroleum	457081	51	0.62	0.84
Alcan Aluminium (AL)	Metals	112193	207	0.61	0.78	Arch. Dan. Midl. (ADM)	Food	468148	50	0.63	0.94
Unilever (UN)	Food & Retail	113736	203	0.58	0.7	Bell Atlantic (BEL)	Telephone Comms.	499768	47	0.63	0.94
Union Electric (UEP)	Electric Services	119737	193	0.6	0.77	Pacific Telesis (PAC)	Telephone Comms.	508091	46	0.63	1.03
Hercules (HPC)	Chemicals	123618	187	0.58	0.84	Lilly Eli & Co. (LLY)	Pharmaceutical	514899	45	0.64	0.91
Air Prod. & Chem. (APD)	Chemicals	123416	187	0.57	0.76	Sara Lee (SLE)	Food & Retail	527814	44	0.63	0.93
Textron (TXT)	Aerospace/Defense	123879	187	0.59	0.75	Dupont (DD)	Chemicals	543724	43	0.62	0.87
Carolina Power&Light (CPL)	Electric Services	131352	177	0.62	0.81	American Express (AXP)	Financial	581840	40	0.65	1
Nortel Networks (NT)	Telephone Apparatus	132384	176	0.61	0.89	Fed. Nat. Mort. (FNM)	Financial	627313	37	0.62	0.88
Baltimore Gas & Elec. (BGE)	Electric Services	142973	163	0.59	0.81	Adv. Micro Dev. (AMD)	Semiconductors	644865	36	0.66	0.99
Hershey Foods (HSY)	Food & Retail	144982	160	0.59	0.82	Citicorp (CCI)	Financial	677484	34	0.65	0.93
Honeywell Int. (HON)	Aerospace/Defense	156376	149	0.6	0.86	Abbott Labs. (ABT)	Pharmaceutical	691877	34	0.63	0.87
Navistar Int. (NAV)	Automotive	168951	138	0.64	0.97	Pfizer (PFE)	Pharmaceutical	689705	34	0.62	0.85
Campbell Soup (CPB)	Food & Retail	175869	132	0.6	0.85	Texas Instruments (TXN)	Semiconductors	708329	33	0.63	0.9
Raytheon (RTN)	Aerospace/Defense	176148	132	0.58	0.79	Boeing Aerospace (BA)	Aerospace/Defense	728779	32	0.64	0.93
United Tech. (UTX)	Aerospace/Defense	190049	122	0.59	0.82	Exxon (XON)	Petroleum Refining	750298	31	0.62	0.91
Nucor (NUE)	Steelworks	194532	119	0.58	0.9	Johnson & Johnson (JNJ)	Pharmaceutical	1001549	23	0.64	0.9
Barnett Banks (BBI)	Financial	202774	115	0.6	0.84	Hewlett-Packard (HWP)	Hardware	1094829	21	0.64	0.92
Phelps Dodge (PD)	Metal Refining	203834	114	0.6	0.85	Home Depot (HD)	Retail	1103037	21	0.65	1.04
McDonnell Douglas (MD)	Aerospace/Defense	203845	114	0.6	0.88	Brist. Myers Squibb (BMY)	Pharmaceutical	1121714	21	0.65	0.88
Fluor (FLR)	Construction	205913	113	0.59	0.83	General Motors (GM)	Automotive	1130452	21	0.66	0.95
General Mills (GIS)	Food & Retail	227318	103	0.59	0.83	Compaq Computer (CPQ)	Hardware	1184985	20	0.67	0.96
Newmont Mining (NEM)	Mining	232391	100	0.64	0.82	Chrysler (C)	Automotive	1231979	19	0.67	0.95
Anheuser Busch (BUD)	Food & Retail	251972	93	0.6	0.88	Coca Cola (KO)	Food & Retail	1244660	19	0.66	0.98
USX-US Steel Grp. (X)	SteelWorks	252435	92	0.61	0.92	Ford (F)	Automotive	1260730	19	0.65	0.92
Alza (AZA)	Pharmaceutical	257116	91	0.62	0.92	GTE (GTE)	Telephone Comms.	1268523	18	0.65	0.91
Alcoa (AA)	Metal Refining	260980	89	0.61	0.78	Pepsico (PEP)	Food & Retail	1321427	18	0.65	1.04
Bank Boston (BKB)	Financial	262506	89	0.63	0.89	General Electric (GE)	Food & Retail	1374682	17	0.63	0.91
Colgate Palmolive (CL)	Food & Retail	262896	88	0.6	0.93	Philip Morris (MO)	Food & Retail	1527659	15	0.66	1.06
Goodyear Tire & Rub. (GT)	Automotive	272025	85	0.61	0.89	IBM (IBM)	Hardware	1677319	14	0.68	0.92
Niagara Mohawk Pow. (NMK)	Electric Services	276284	84	0.62	1.07	AT&T (T)	Telephone Comms.	1689767	14	0.66	1.04
Atlantic Richfield (ARC)	Petroleum	286580	81	0.6	0.76	Wal Mart (WMT)	Retail	1794160	13	0.7	1
FPL Group (FPL)	Electric Services	303364	77	0.62	0.93	Merck & Co. (MRK)	Pharmaceutical	2055443	11	0.69	0.93
Royal Dutch Petrol. (RD)	Petroleum	304505	76	0.6	0.78	Motorola (MOT)	Hardware	2204059	11	0.67	1.08

Companies range in average market capitalisation from 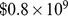 to 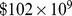 over the period, and are ranked in order of decreasing average value of ITT (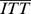). We include all trades occurring during NYSE trading hours (9.30am–4pm EST), excluding public holidays and weekends.

We also analyse one hundred NASDAQ stocks from fourteen industry sectors: Technology-Hardware(28), Technology-Software(16), Semiconductors(7), Pharmaceutical, Biotechnology & Medical Equipment(12), Financial(5), Automotive(1), Steel Works(1), Chemicals(1), Retail & Food(16), Petroleum, Gas & Heavy Machinery (2), Telephone & Cable Television Service Providers(5), Services(2), Transportation(3), Electrical Apparatus(1). We study the time intervals between successive stock trades as recorded in the TAQ database, for twenty-nine companies over the period 4 Jan. 1993–31 Dec. 1996, and seventy one companies over the period 3 Jan. 1994–30 Nov. 1995 (marked with (*) in Table 2). For both markets, we select companies with average market capitalisations ranging over three decades, and varying levels of trading activity with average values of intertrade time between 11 and 640 seconds for NYSE stocks, and between 5 and 680 seconds for NASDAQ stocks. In parallel with the intertrade times, we analyse the prices for both sets of stocks over the same periods.

**Table 2:** Characteristics of one hundred NASDAQ stocks studied; data covers twenty nine companies over the period 4 Jan. 1993–31 Dec. 1996, and seventy one companies (marked with *) over the period 3 Jan. 1994–30 Nov. 1995.

Company Name(Ticker Symbol)	Industry	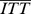 Numberof Trades	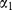 (sec)	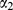 ITT	ITT	Company Name(Ticker Symbol)	Industry	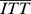 Numberof Trades	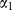 (sec)	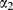 ITT	ITT
Oshkosh B Gosh (GOSHA)	Retail & Food	31986	683	0.68	0.73	US Robotics (USRX*)	Hardware	143912	78	0.75	0.8
Sanmina-SCI (SANM*)	Hardware	22648	438	0.7	0.81	Symantec (SYMC*)	Software	143405	77	0.77	0.75
MedImmune (MEDI*)	Biotech.	24618	414	0.66	0.97	Autodesk (ACAD)	Software	261716	74	0.77	0.83
ICOS (ICOS*)	Pharmaceutical	32460	339	0.65	0.87	Oxf. Health Plans (OXHP*)	Financial	150501	74	0.75	0.87
Gilead Sciences (GILD*)	Biotech.	32187	332	0.67	0.83	Komag (KMAG*)	Hardware	175953	63	0.79	0.77
Molex (MOLX*)	Hardware	34104	321	0.7	0.66	Biomet (BMET)	Med. Apparatus	379342	62	0.73	0.94
Coors Adolph (ACCOB)	Food & Retail	78393	295	0.67	0.73	Novellus Systems (NVLS*)	Hardware	182185	61	0.76	0.77
Whole Foods Mar. (WFMI*)	Food & Retail	38018	290	0.69	0.89	Mobile Tel. Tech. (MTEL*)	Telephone Comms.	184469	61	0.8	0.93
Ross Stores (ROST*)	Food & Retail	42772	256	0.7	0.85	KLA-Tencor (KLAC*)	Hardware	187614	60	0.78	0.71
XOMA (XOMA*)	Pharmaceutical	43073	256	0.68	0.91	St. Jude Medical (STJM)	Med. Apparatus	388393	59	0.79	0.82
Paccar (PCAR)	Automotive	94496	245	0.72	0.77	AST Research (ASTA*)	Hardware	191683	58	0.76	0.91
General Nutr. Cos. (GNCI*)	Food & Retail	48222	226	0.71	0.79	Parametric Tech. (PMTC*)	Software	197637	57	0.79	0.83
Ryans Fam. Steak. (RYAN)	Food & Retail	108243	215	0.66	0.77	Starbucks (SBUX*)	Food & Retail	201225	56	0.79	0.86
Caliber System (ROAD)	Transportation	106570	209	0.72	0.81	Read-Rite (RDRT*)	Hardware	205021	54	0.76	0.8
Giddings & Lewis (GIDL)	Heavy Machinery	57081	204	0.7	0.83	Borland Software (BORL*)	Software	207697	54	0.75	0.97
Huntington Banc. (HBAN*)	Financial	55885	199	0.68	0.83	Gateway 2000 (GATE*)	Hardware	217267	52	0.8	0.89
Worthington Ind. (WTHG)	Steelworks	119751	194	0.7	0.69	LM Ericsson Tel. (ERICY*)	Hardware	228287	49	0.73	0.95
Phycor (PHYC*)	Office Services	59431	183	0.75	0.76	StrataCom (STRM*)	Hardware	235537	48	0.77	0.8
Intergraph (INGR)	Hardware/Software	131780	176	0.7	0.87	Xilinx (XLNX*)	Semiconductors	239423	47	0.76	0.79
Shared Medical Sys. (SMED)	Hardware/Software	132579	175	0.76	0.8	Biogen (BGEN*)	Biotech.	241886	46	0.82	0.9
Glenayre Tech. (GEMS*)	Hardware	63152	174	0.69	0.81	Adaptec (ADPT*)	Hardware	253082	44	0.78	0.75
PETsMART (PETM*)	Food & Retail	67047	165	0.72	0.83	Acclaim Ent. (AKLM*)	Software	282481	40	0.8	0.92
Tyson Foods (TYSNA*)	Food & Retail	70711	158	0.7	0.79	Chiron (CHIR*)	Pharmaceutical	292353	38	0.81	0.85
MFS Comms. (MFST*)	Telephone Comms.	70776	157	0.73	0.86	Tellabs (TLAB*)	Hardware	299490	38	0.79	0.84
Brunos (BRNO)	Food & Retail	99211	155	0.67	0.88	Adobe Systems (ADBE*)	Software	307959	36	0.8	0.87
Sigma-Aldrich (SIAL*)	Chemicals	72843	153	0.69	0.89	America Online (AMER*)	Services	314541	36	0.74	0.94
Atlantic S.E. Air. (ASAI*)	Transportation	75031	148	0.72	0.85	Electronic Arts (ERTS*)	Software	329541	34	0.79	0.81
Cephalon (CEPH*)	Pharmaceutical	71733	148	0.7	0.88	Qualcomm (QCOM*)	Hardware	335494	34	0.77	0.87
Safeco (SAFC)	Financial	157461	148	0.77	0.78	Informix (IFMX*)	Software	350185	32	0.8	0.82
Comcast (CMCSA)	Cable TV	161408	144	0.72	0.79	Altera (ALTR*)	Semiconductors	349925	32	0.78	0.88
Stew. & Stev. Svcs (SSSS*)	Heavy Machinery	79177	141	0.75	0.81	Tele Comms. (TCOMA)	Cable TV	765301	31	0.72	0.98
American Greetings (AGREA)	Food & Retail	169265	138	0.73	0.76	Amer. Pow. Conv. (APCC*)	Electrical Apparatus	395510	28	0.71	1.02
Northwest Airlines (NWAC*)	Transportation	77658	127	0.76	0.88	Lotus Devel. (LOTS)	Software	582256	25	0.82	0.97
ADC TeleComms. (ADCT*)	Hardware	90573	123	0.74	0.77	Integr. Dev. Tech. (IDTI*)	Semiconductors	471169	24	0.79	0.86
Charming Shoppes (CHRS)	Food & Retail	196473	119	0.71	0.89	Cirrus Logic (CRUS*)	Semiconductors	500710	22	0.79	0.8
HBO & Co. (HBOC*)	Hardware/Software	95662	116	0.78	0.71	US HealthCare (USHC*)	Financial	505215	22	0.76	0.97
Microchip Tech. (MCHP*)	Semiconductors	102625	109	0.73	0.8	MCI Comms. (MCIC)	Telephone Comms.	1096316	21	0.74	0.94
Andrew (ANDW)	Hardware	215063	109	0.72	0.79	DELL (DELL*)	Hardware	557195	20	0.8	0.82
Legent (LGNT*)	Software	90705	108	0.75	0.92	DSC Comms. (DIGI)	Hardware	1209063	19	0.79	0.84
Stryker (STRY*)	Medical Apparatus	107678	104	0.79	0.8	Applied Materials (AMAT*)	Hardware	584276	19	0.79	0.79
PeopleSoft (PSFT*)	Software	108433	102	0.76	0.79	Sybase (SYBS*)	Software	631753	18	0.79	0.98
Outback Steak. (OSSI*)	Food & Retail	112607	99	0.75	0.86	Amgen (AMGN)	Biotech.	1392229	17	0.79	0.91
Boatmens Banc. (BOAT)	Financial	236139	99	0.73	0.81	3Com (COMS*)	Hardware	699889	16	0.79	0.77
Intelligent Elec. (INEL*)	Hardware	113666	98	0.74	0.89	Apple Computer (AAPL)	Hardware	1646925	14	0.76	0.97
Genzyme General (GENZ*)	Biotech.	116223	96	0.76	0.83	Novell (NOVL)	Software	1803407	13	0.74	1.04
Bed Bath & Beyond (BBBY*)	Food & Retail	120723	92	0.79	0.8	Oracle (ORCL)	Software	1817365	13	0.77	0.89
Intuit (INTU*)	Software	122051	91	0.74	1.03	Sun Microsystems (SUNW)	Hardware/Software	2029156	12	0.79	0.81
Boston Chicken (BOST*)	Food & Retail	128376	87	0.74	0.91	Cisco Systems (CSCO*)	Hardware	1093386	10	0.77	0.95
Staples (SPLS*)	Food & Retail	132041	85	0.78	0.78	Microsoft (MSFT*)	Software	1505531	7	0.77	0.78
Linear Tech. (LLTC*)	Semiconductors	139953	80	0.77	0.84	Intel (INTC)	Semiconductors	4807756	5	0.77	0.89

Companies range in average market capitalisation from 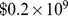 to 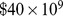, and are ranked in order of decreasing average value of ITT (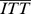). We include all trades occurring during regular NASDAQ trading hours (9.30am–4pm EST), excluding public holidays and weekends.

## Method

Like many financial time series the intertrade times (ITT) are inhomogeneous and nonstationary, with statistical properties changing with time, e.g. ITT data exhibit trends superposed on a pattern of daily activity [24]. While ITT fluctuate in an irregular and complex manner on a trade-by-trade basis, empirical observations reveal that periods of inactive trading are often followed by periods of more active trading (Fig. 1). Such patterns can be seen at scales of observation ranging from minutes to months, suggesting that there may be a self-similar, fractal structure in the temporal organisation of intertrade times, independent of the average level of trading activity of a given stock [24].

To probe for scale-invariant features in the fluctuations of intertrade times, we apply the detrended fluctuation analysis (DFA) method, which has been shown to detect and accurately quantify long-range power-law correlations embedded in noisy non-stationary time series with polynomial trends [25]. We choose this method because traditional techniques such as power spectral, autocorrelation and Hurst analyses are not suited to nonstationary data [26]. The DFA method (DFA-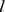) quantifies the root-mean-square fluctuations 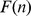 of a signal at different time scales 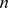, after accounting for nonstationarity in the data by subtracting underlying polynomial trends of order (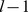). A power-law functional form 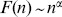 indicates self-similarity and fractal scaling in the ITT time series. The scaling exponent 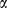 quantifies the strength of correlations in the ITT fluctuations: if 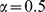 there are no correlations, and the signal is uncorrelated random noise; if 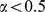 the signal is anti-correlated, meaning that large values are more likely to be followed by small values; if 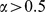 there are positive correlations and the signal exhibits persistent behaviour, where large values are more likely to be followed by large values and small values by small values. The higher the value of 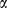, the stronger the correlations. The DFA method avoids the spurious detection of apparent long-range correlations that are an artifact of polynomial trends and other types of nonstationarities [27]–[30].

## Results

**Figure 2:**
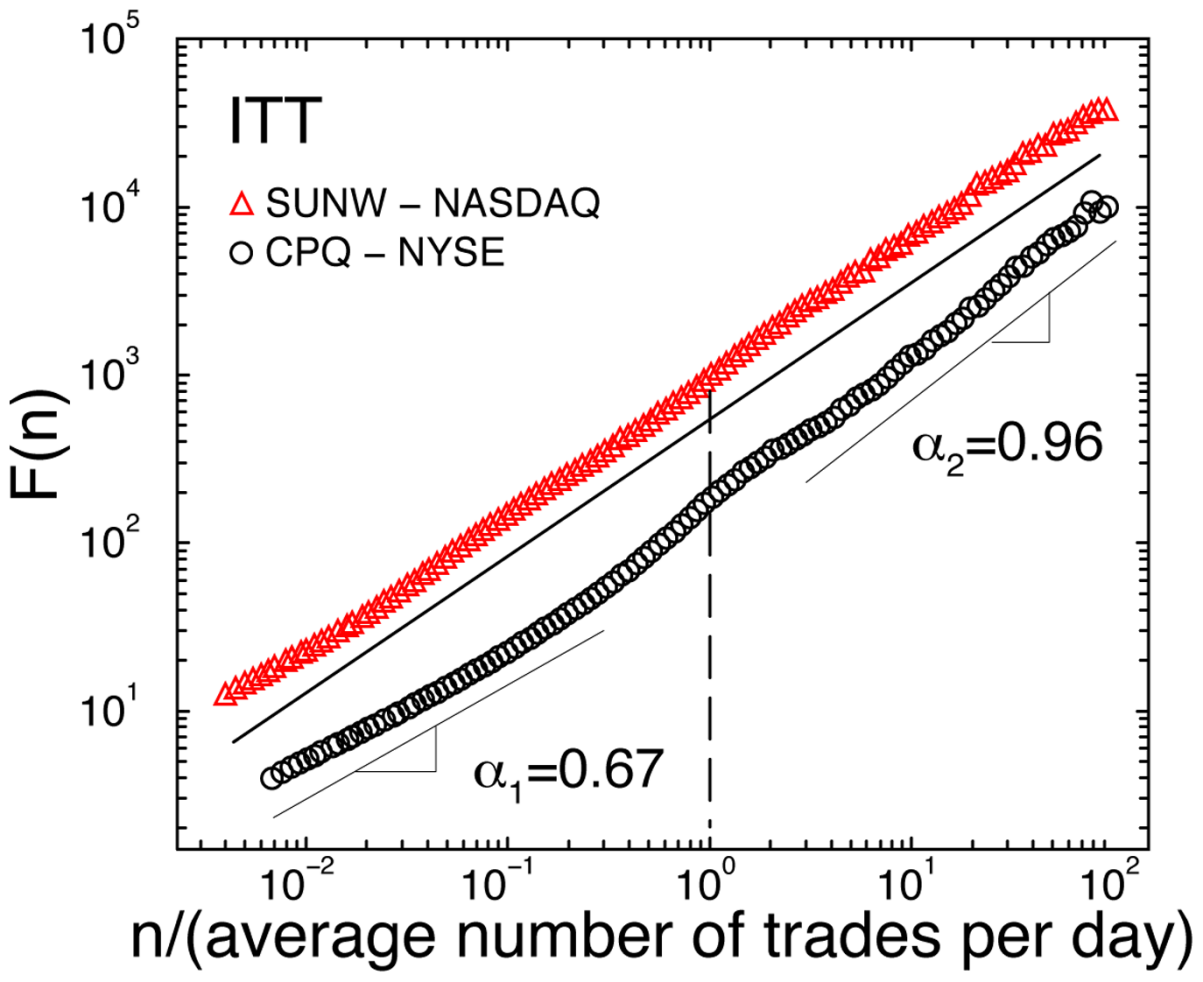
Root-mean-square fluctuation function 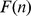 obtained using DFA-2 analysis, for the intertrade times (ITT) of stock in NASDAQ company Sun Microsystems (SUNW) and NYSE company Compaq Computer Corp. (CPQ). Here 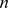 indicates the time scale in number of trades. We normalize the time scale 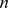 by the daily average number of trades for each stock, so that a unit normalized scale indicates one trading day (marked by a dashed line). The scaling curves are vertically offset for clarity. While both companies have similar market capitalisations, industry sectors and average levels of trading activity (average ITT) and exhibit long-range power-law correlations over a broad range of scales, the scaling behaviour of the intertrade times for the two stocks is quite different. For CPQ we find a pronounced crossover from weaker correlations over time scales smaller than a day, to stronger correlations over time scales larger than a trading day (
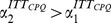
). In contrast, the scaling function 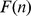 for SUNW does not exhibit such a crossover, and we find much stronger correlations over time scales smaller than a trading day compared with CPQ (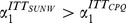).

**Figure 3:**
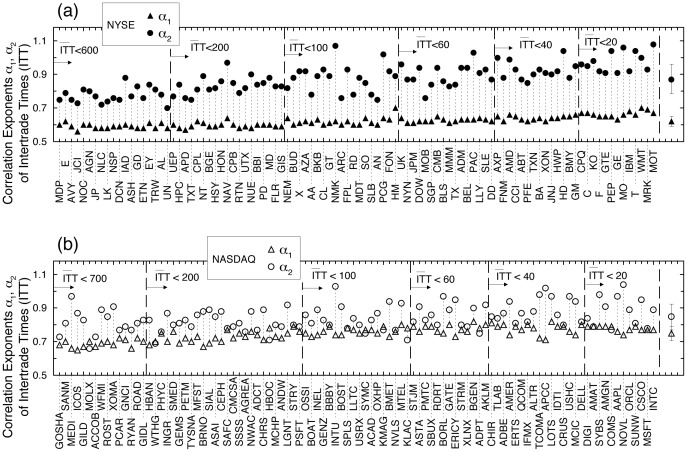
Different correlation properties in intertrade times for stocks registered on the NYSE and NASDAQ markets. Correlation exponents 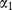 and 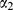 characterising the temporal structure in ITT for (a) one hundred NYSE stocks and (b) one hundred NASDAQ stocks, of companies with a broad range of market capitalisations and industry sectors. Stocks are ranked in order of decreasing average value of ITT (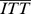) (as in Tables 1 and 2), and are split into subsets (marked by vertical dashed lines) of companies with matching 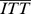, and with approximately equal number of stocks in each subset. We estimate 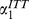 over scales from 8 trades to half of the daily average number of trades (for stocks with fewer than 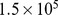 trades/year), and to a third of the daily average number of trades (for stocks with more than 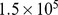 trades/year). We estimate 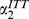 over scales from 3 to 100 times the daily average number of trades. Group averages and standard deviations of 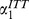 and 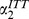 are shown to the right of the panel for each market. Systematically higher values of 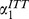 for the NASDAQ stocks as compared to the NYSE stocks (statistically significant difference with p-value 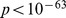 by Student's t-test), suggest an underlying influence of market structure on the temporal organisation of intertrade times over scales within a trading day. In contrast, no systematic differences between the two markets are observed in the values of 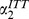, characterising correlation properties of intertrade times over scales above a trading day (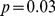 by Student's t-test). We find similar results when we analyse trading activity at high resolution in terms of the number of trades per minute: a crossover at one trading day and stronger correlations for NASDAQ stocks compared to NYSE stocks over time scales less than a day (features which were not observed in previous studies [52], [50]). We further observe an increasing trend in the values of 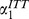 and 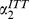 with decreasing 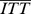 and increasing company capitalisation for the companies on both markets.

We find that the ITT series for all stocks on both markets exhibit long-range power-law correlations over a broad range of time scales, from several trades to hundreds of thousands of trades, characterised by a scaling exponent 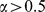 (Fig. 2 and Fig. 3). For all stocks on both markets we observe a crossover in the scaling curve 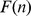 from a scaling regime with a lower exponent 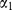 over time scales less than a trading day, to a scaling regime with an exponent 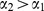 (stronger positive correlations) over time scales from days to almost a year.

Further, we find that this crossover is systematically more pronounced for NYSE stocks compared to NASDAQ stocks (Fig. 2 and Fig. 3). Characterising ITT fluctuations over time scales less than a day, we find that NASDAQ stocks exhibit statistically stronger correlations than NYSE stocks as indicated by Student's t-test (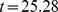, 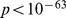), with significantly higher average value of the exponent 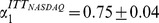 (group mean 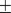 std. dev.) as compared to 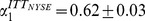 (Fig. 3). In contrast, over time horizons above a trading day, we find that the correlation properties of ITT on both markets are statistically similar (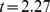, 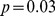), with average scaling exponent 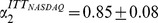 comparable with 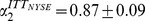 (Fig. 3). Values for the scaling exponents 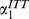 and 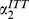 for the companies on the NYSE and NASDAQ markets are shown in Table 1 and Table 2 respectively.

**Figure 4:**
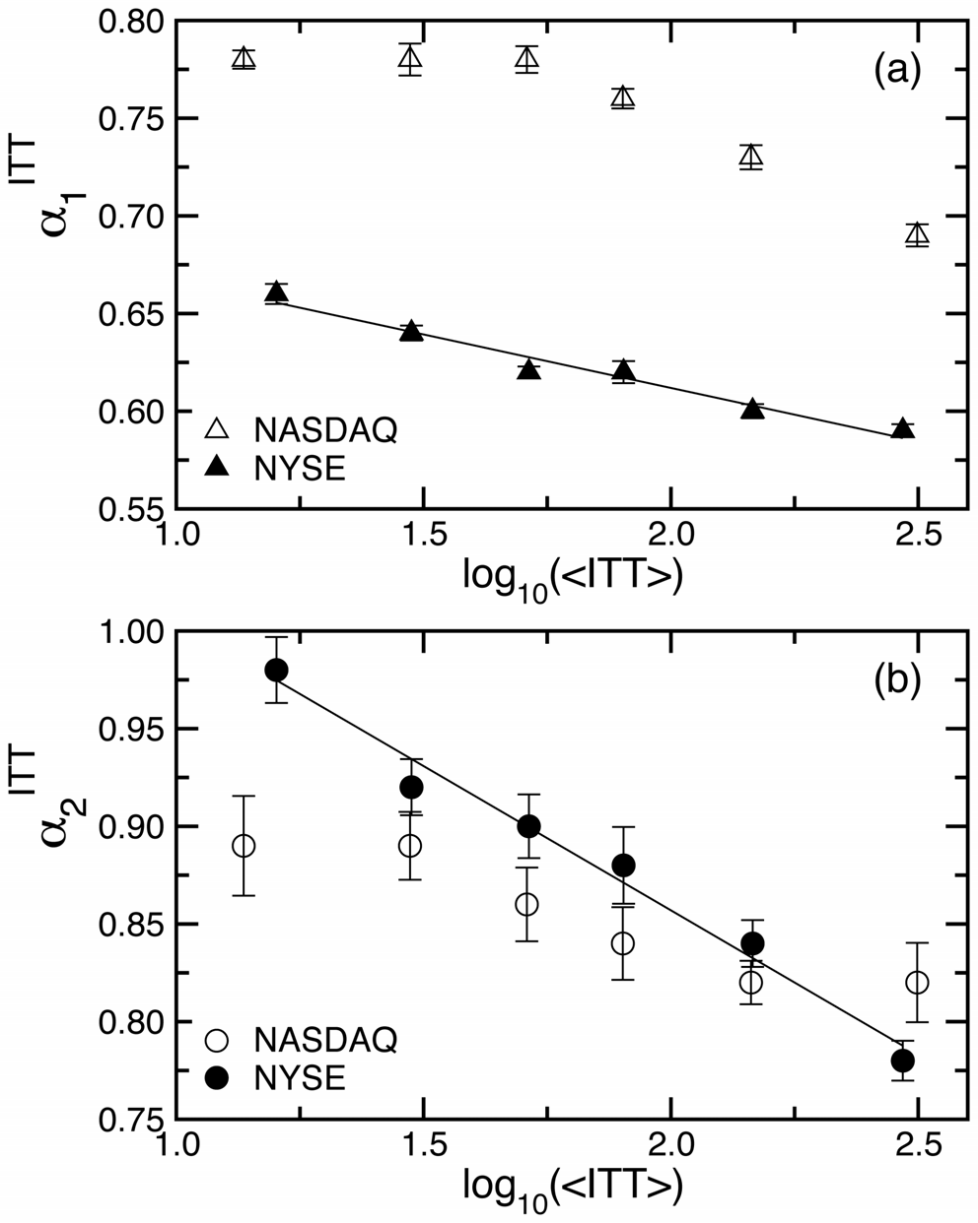
Comparing long-range correlations in ITT for groups of stocks with varying average levels of trading activity on the NYSE and the NASDAQ. (a) Dependence of exponent 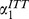, characterizing the strength of correlations in ITT over scales from seconds up to a trading day, on the average level of trading activity. Each datapoint represents the group average over a subset of stocks, with a matching range of average intertrade times 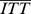 for the two markets. Stocks are grouped into subsets as indicated by vertical dashed lines in Fig. 3a,b. The consistent difference in the scaling exponent 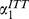 between NYSE and NASDAQ stocks suggests that independent of company characteristics such as market capitalization and industry sector, the temporal organization of ITT within a trading day carries an imprint of market structure. (b) Dependence of exponent 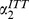 characterizing correlations in ITT over time scales from a trading day to several months, on the average level of trading activity. On both markets we observe similar behavior with no systematic difference in the values of 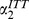 between NYSE and NASDAQ subsets of stocks with matching ranges of 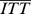. These results suggest that over time horizons longer than a trading day, the impact of market structure on trading dynamics is less pronounced as more information is available to investors over longer time scales, driving their trading activity. The resulting more coherent behavior of investors is reflected in stronger correlations 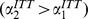 over longer time scales.

We next investigate how the correlation properties of ITT depend on the average level of trading activity, and if this dependence differs with market structure. Since both sets of a hundred stocks that we study on the NYSE and NASDAQ markets encompass a range of average trading activity spanning over two decades, we split both sets into six subsets with matching average ITT (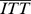) and approximately equal numbers of stocks in each subset (Fig. 3a,b). Within each market we find that over time scales less than a day, the correlation exponent 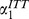 characterising the trading dynamics is larger for stocks with higher trading activity (lower 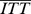) and correspondingly higher market capitalisation (Fig. 3a,b and Fig. 4a). Surprisingly, this dependence persists also for 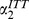, characterizing the dynamics over much longer time scales, ranging from days to months (Fig. 4b). For NYSE stocks we find a logarithmic dependence of 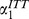 and 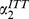 on 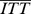 (subsequent to posting this manuscript on the Los Alamos archive [31], this logarithmic dependence was later confirmed in [32] on a different set of NYSE stocks). This dependence does not appear to hold for NASDAQ stocks (Fig. 4).

We then compare the scaling behaviour of ITT for each subset of NASDAQ stocks with the corresponding subset of NYSE stocks with matching 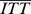. We find that for each subset the average correlation exponent 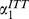 for the NASDAQ stocks is significantly higher compared to the NYSE stocks (all 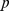 values 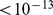; Fig. 4a). We also find that there is no significant correlation between the differences 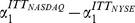 in each subset and the 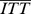, as indicated by Pearson's test (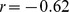, 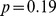). These observations show that within a trading day the difference in the correlation properties of intertrade times of NYSE and NASDAQ stocks is independent of the average level of trading activity. In contrast to 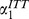, there is no systematic difference in the values of the average 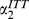 for NASDAQ and NYSE stocks for subsets with matching 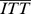 (all 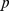 values 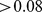; Fig. 4b) except for the subset of companies with the highest frequency of trading (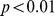; Fig. 4b).

Since for both NYSE and NASDAQ stocks we have chosen companies representing eleven industry sectors with a broad range of market capitalisations and average levels of trading activity spanning over more than two decades, our findings of (i) a crossover in the scaling behaviour of ITT that is more pronounced for NYSE stocks, and (ii) stronger correlations over intraday time scales of NASDAQ stocks with higher values for 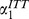 compared to NYSE stocks, support our hypothesis that market structure affects the dynamics of transaction timing. However, more established companies listed on the NYSE may be subject to different trading patterns when compared with the younger and more rapidly growing companies on the NASDAQ. To verify that the stronger correlations in ITT over time scales less than a day for NASDAQ stocks are indeed due to market structure, we ask if the scaling properties of ITT systematically change for companies that transfer from the NASDAQ to the NYSE. In particular, we investigate the trading dynamics of ten companies that moved from the NASDAQ to the NYSE around the end of 1994 and the beginning of 1995 (Table 3). For each company, we analyse the ITT time series while the company was registered on the NASDAQ, and then repeat the analysis when the company was on the NYSE.

**Table 3:** Characteristics of ten stocks that moved from the NASDAQ to the NYSE during the period 3 Jan. 1994–30 Nov. 1995.

Company	Industry	NASDAQ				NYSE			
		TickerSymbol	Numberof Days	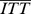 Numberof Trades	(sec)	TickerSymbol	Numberof Days	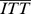 Numberof Trades	(sec)
Input Output	Measuring Devices	IPOP	219	25211	198	IO	265	10944	540
Consolidated Papers	Paper Mills	CPER	154	8902	389	CDP	330	15180	488
Cardinal Health	Wholesale Drugs	CDIC	171	11510	333	CAH	313	14819	475
AK Steel Holding Corp.	Steelworks	AKST	256	14575	383	AKS	167	10397	364
Sports & Recreation	Retail	SPRC	177	17721	222	WON	307	19907	345
State Street Boston	Financial	STBK	282	43829	148	STT	202	16916	273
Dollar General	Retail	DOLR	273	34873	180	DG	211	19817	241
Mid-Atlantic Medical Services	Financial	MAMS	187	90598	48	MME	297	50245	136
Seagate	Hardware	SGAT	238	119544	46	SEG	246	85100	67
Newbridge Networks	Hardware	NNCXF	176	208771	20	NN	308	148637	28

Companies are ranked in order of decreasing average value of ITT when on the NYSE. We include all trades occurring during NYSE trading hours (9.30am–4pm EST) excluding public holidays and weekends.

**Figure 5:**
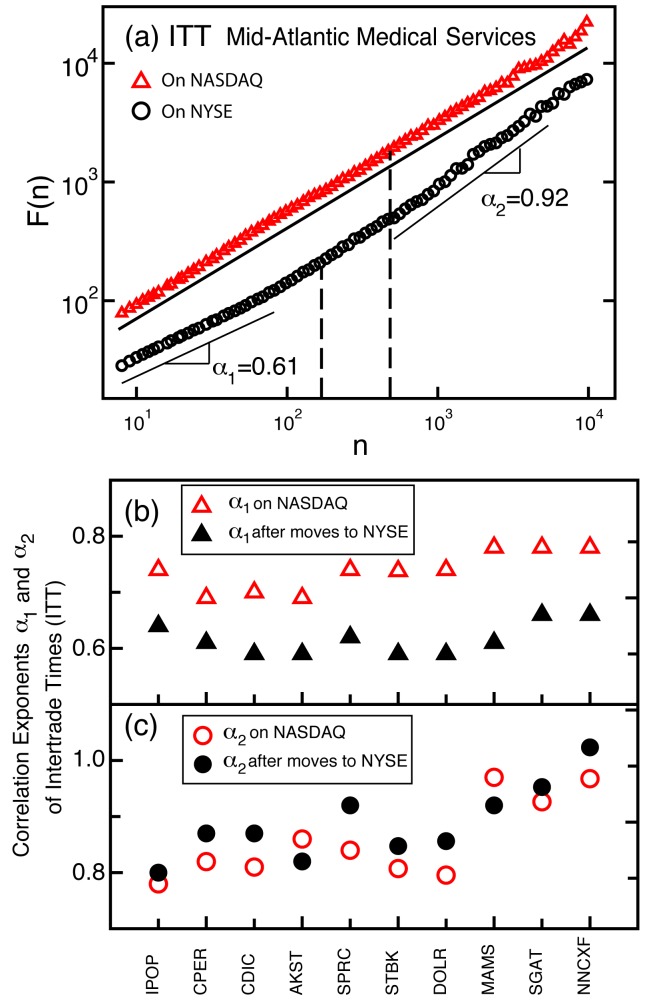
Correlation properties of intertrade times of companies that moved from the NASDAQ to the NYSE. (a) Fluctuation function 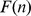, obtained using DFA-2 analysis on ITT of stock in the company Mid-Atlantic Medical Services Inc. while it was on the NASDAQ (3 Jan. 1994–29 Sep. 1994) and then after it moved to the NYSE (30 Sep. 1994–30 Nov. 1995). Here 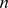 indicates the scale in number of trades and the vertical dashed lines indicate the average daily number of trades while on the NYSE or the NASDAQ. The two scaling curves are vertically offset for clarity. After the move to the NYSE there is a decrease in the correlation exponent 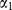 at time scales within a trading day and a pronounced crossover to stronger correlations with a higher exponent 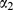 at larger time scales. (b) 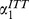 characterising fluctuations over time scales less than a trading day in ITT of stock in ten companies that moved from the NASDAQ to the NYSE. Companies are ranked in order of decreasing 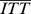 while on the NYSE (as in Table 3) and the scaling range for 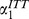 is the same as for the hundred NYSE and NASDAQ stocks (Fig. 3a,b). For all companies there is a decrease in 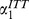 after the move to the NYSE, indicating that the transition to weaker correlations in ITT over time scales less than a day is due to the NYSE market structure and not to company-specific characteristics. (c) 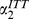 over time scales extending from a trading day to almost a year. In this case we do not observe any systematic change when companies move to the NYSE, which is consistent with the finding of statistically similar values of scaling exponent 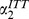 for the two groups of the one hundred stocks registered on the NYSE and on the NASDAQ (Fig. 3a,b).

For all ten companies we find a significant change in the scaling properties of intertrade times: a marked decrease in the strength of the power-law correlations within a trading day (lower 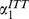) associated with the transfer from the NASDAQ to the NYSE (average difference 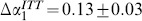; Fig. 5b). There is however, no corresponding systematic change in the correlations over time scales above a trading day (average difference 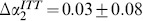; Fig. 5c), consistent with our findings of statistically similar values of scaling exponent 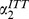 for the two groups of one hundred stocks registered on the NYSE and NASDAQ (Fig. 2 and Fig. 3). Thus, our results indicate that market structure impacts not only trading dynamics on a trade-by-trade basis [19], but also the fractal temporal organisation of trades over time scales from a few seconds up to a day. The presence of stronger intraday correlations in transaction timing for NASDAQ stocks may be attributed to the multiplicity of dealers (ranging from 2 to 50 per stock during 1994 [11]) and electronic methods of trading (Electronic Communication Networks and the Small Order Execution System [7]), allowing the NASDAQ to efficiently absorb fluctuations in trading activity in almost real time [5]. In contrast, for each stock on the NYSE, while there is the electronic SuperDOT routing system, each order has to be exposed to and compared with outstanding orders, as the single NYSE specialist finds the best bid to match an offer with [6]. This may lead to interruptions in the execution of a rapid succession of trades on the NYSE, resulting in weaker correlations in intertrade times within a trading day.

On the other hand, our finding of stronger power-law correlations for both markets over time horizons from a trading day to several months (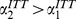) suggests that investors' behaviour is more coherent over longer time scales, as information driving trading activity takes time to disseminate. Moreover, this can account for the similar values of 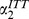 for subsets of NYSE and NASDAQ stocks with matched 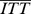 (Fig. 4b), since news and information driving trading activity are exogenous to market structure.

**Figure 6:**
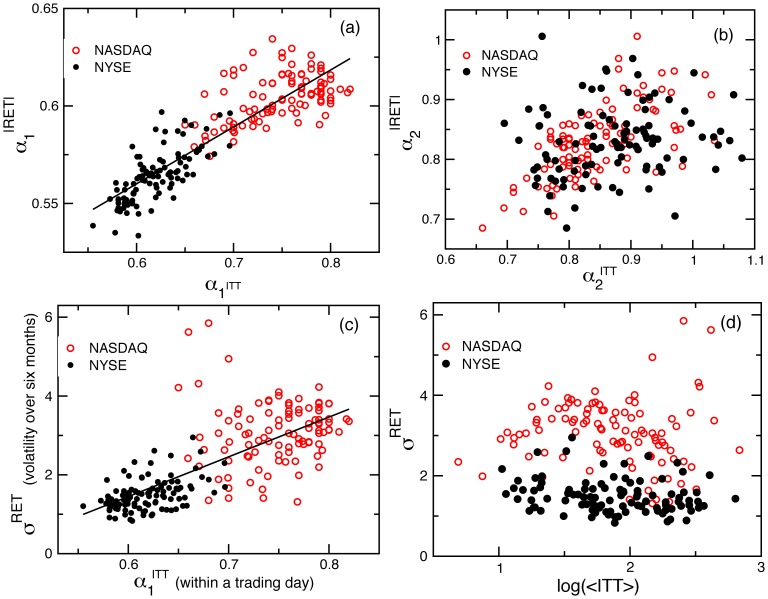
Relation between correlations in intertrade times and stock price dynamics. (a) Dependence of exponent 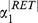 characterising power-law correlations in absolute logarithmic price return fluctuations, on correlation exponent 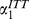 characterising intertrade times within a trading day. Data represent one hundred NYSE (Table 1) and one hundred NASDAQ (Table 2) stocks. We calculate price returns over 1-minute intervals and 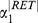 over time scales from 8 to 180 minutes (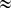 half a trading day, which is 390 minutes). The positive relationship between 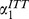 and 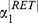 indicates that stronger correlations in ITT are coupled with stronger correlations in price fluctuations. This finding suggests that price fluctuations are not merely a response to short-term bursts of trading activity [34], [16]: rather the fractal organisation of price fluctuations over a broad range of time scales is linked to the observed underlying scaling features in the series of intertrade times. (b) Strong relationship between correlations in ITT and correlations in price fluctuations over time scales larger than a trading day for NASDAQ stocks. In contrast, there is no corresponding positive relationship for NYSE stocks. This suggests a weaker coupling between trading dynamics and price formation under the NYSE market structure, over time horizons above a trading day. Dependence of stock price volatility 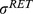 on (c) the correlation exponent 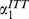 and (d) the average value of ITT for the same stocks as in (a). We calculate 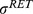 as the standard deviation of daily logarithmic price returns over six-month periods, averaging over all six-month periods throughout the entire record of each stock. Our results show no strong dependence between stock price volatility 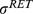 and average level of trading activity, rather the volatility appears sensitive to the strength of the temporal correlations in ITT. These findings suggest that scale-invariant features in transaction times may play an important role in price formation. Furthermore, both dynamic and static properties of stock prices appear to be influenced by market-specific features in transaction timing: stronger power-law correlations in ITT (higher values of 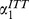) for NASDAQ stocks are matched by stronger power-law correlations in price fluctuations (higher values of 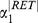) and higher volatility (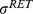), compared with NYSE stocks.

Finally, we investigate if the market-mediated differences in long-range power-law correlations in ITT translate into differences in the scaling behaviour of price fluctuations of stocks registered on the NASDAQ and NYSE markets. To this end, in parallel with ITT we analyse the absolute price returns for each company in our database for both markets. For all stocks we observe a crossover at a trading day in the scaling function 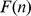 of price fluctuations [33], [24], from weaker to stronger correlations, corresponding to the crossover we observe for intertrade times. In addition we find that over time scales less than a day, stocks with stronger correlations in ITT exhibit stronger correlations in absolute price returns (Fig. 6a), as indicated by Pearson's test (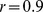, 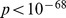). In particular, we find that the stronger correlations in ITT associated with the NASDAQ market structure (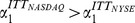), are accompanied by stronger correlations in price fluctuations (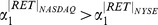) over time scales within a trading day (Fig. 6a).

We also find evidence of a positive relationship between correlations in ITT and correlations in price fluctuations over time scales larger than a trading day for NASDAQ stocks (Pearson's test shows statistically significant correlation between 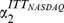 with 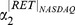 with 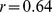, 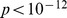; Fig. 6b). In contrast, there is no corresponding positive relationship between 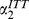 with 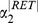 for NYSE stocks (Pearson's test: 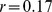, 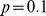), suggesting a weaker coupling between trading dynamics and price formation under the NYSE market structure, over time horizons above a trading day. While previous work has suggested that bursts of trading activity have an instantaneous impact on stock prices [19], [34], our results show that the interaction between trading times and price formation is more complex, where scale invariant temporal patterns in ITT are linked with scaling features of price fluctuations over a broad range of time scales.

We then test whether long-range correlations in ITT are also linked with stock price volatility. Previous studies have reported higher price volatility for NASDAQ stocks compared to NYSE stocks [4], [9], [10]. We find a positive relationship, with stronger correlations in ITT over time scales less than a day related to higher daily volatility 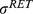 (Pearson's test: 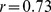, 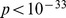; Fig. 6c). Further, we find that the NASDAQ stocks have higher 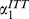 and correspondingly higher 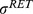 compared to NYSE stocks (Fig. 6c). This relationship may appear to follow from our observation that 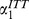 depends on 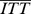 (Fig. 4a), and previous studies which connect price volatility with periods of high transaction rates [16], [35]. However, for the stocks in our database (Tables 1 and 2), we find no correlation between 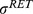 and average level of trading activity as measured by 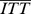 (Pearson's test: 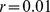, 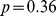; Fig. 6d). Thus the relationship between 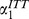 and 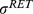 suggests that information contained in the microscopic temporal structure of ITT is carried over a range of scales to impact daily price volatility.

## Discussion

Understanding the statistical properties of intertrade times and the related underlying mechanism is crucial for the development of more realistic models not only of the flow of transactions [36]–[38], but more importantly to elucidate (i) the relation between intertrade time dynamics and stock price formation [16], [18], [39]–[41], and (ii) how the process of stock price formation is influenced by market structure. In that context, several prior studies have focused not only on the correlation properties, but also on nonlinear features of intertrade times, and on the functional form of their probability distribution. Early studies reported stretched exponential distributions for intertrade times based on data from a single actively-traded stock over a short period of a few months [16], [17], or power-law tailed distributions for rarely-traded 19th century stocks [42] and eurobonds traded in 1997 [43]. While some of these studies have also considered autocorrelations in intertrade times, they have not identified the functional form of these correlations and whether they are persistent or anti-persistent. A first systematic empirical study based on 30 frequently-traded US stocks over a long period of several years [24] has (i) reported long-range power-law correlations of persistent type with a characteristic crossover to a superdiffusive behavior at time scales above a trading day, and (ii) identified a Weibull functional form for the distribution of intertrade times. In a follow up study based on a different group of US stocks [38], the Weibull functional form was also considered a good fit for the intertrade time distribution, with the Tsallis q-exponential form as an alternative. Further investigations considering the intertrade dynamics of a group of frequently-traded Chinese stocks have shown that the Weibull distribution outperforms the Tsallis q-exponential for more than 98.5% of the data [20]. The long-range power law correlations in intertrade times initially reported for US stocks [24] were also observed for liquid stocks on the Shanghai Stock Exchange [22]. Our results based on 100 NASDAQ and 100 NYSE stocks confirm the presence the long-range power law correlations. The results of these studies, which focus on different markets and different time periods, confirm that the Weibull distribution and long-range power law correlations are stable characteristics of intertrade time dynamics across markets and temporal time scales. Interestingly, similar characteristics were recently reported for commodity dynamics of ancient Babylon (463–72 B.C), and medieval and early modern England (1209–1914 A.D.) markets [44].

It has been recently hypothesized [36] that the dynamics of intertrade times maybe governed by a priority decision-based queuing mechanism [45], [46]. This hypothesis, however, does not appear plausible. First, the priority queuing process proposed in [45] leads to power law distributions for the timing between events, which has been rejected for intertrade times [20], [38]. Second, this queuing process does not generate long-term correlations, contrary to empirical findings for intertrade times of stocks reported in [20], [22], [24], and in the current study comparing stocks on different markets. Moreover, the activity pattern of a single stock broker is not adequately described by a power law, but rather by a power law with a stretched exponential tail [46], which is actually the functional form of the Weibull distribution [24]. Further, it is unlikely that the priority decision-based queuing process underlies stock market operations, since market agents treat all orders for stock transactions with the same priority no matter how big or small the order, because the objective of market agents is to execute all orders as soon as possible. For this reason, each stock transaction is a minimal time event realization resulting from the competition of a number of market agents with different reaction times–the statistics of minimal events derived from multiple realizations are described by Weibull distributions. Thus, the process of stock market operations is markedly different from the processes governing the dynamics of other human activities, such as web browsing or email exchange that are based on priority queuing [45], [46]. Furthermore, in contrast to priority decision-based processes, intertrade dynamics exhibit nonlinear (multifractral) properties, as first empirically identified in [24] and later confirmed in the framework of multifractal random walks [36].

To summarize, this is the first large empirical study to investigate intertrade times comparing 200 stocks registered on the NYSE and NASDAQ markets representing diverse sectors of the economy, where all stock transactions over a period of four years are included (Table 1 and 2, Figure 2 and Figure 3). This is also the first study to examine changes in the trading dynamics of stocks of companies that moved from one market to the other (Table 2 and Figure 5).

We report that trading dynamics of company stocks are characterized by a scale-invariant temporal organisation of intertrade times which is significantly different for stocks registered on the NYSE and the NASDAQ, indicating that market structure influences the correlation properties of transaction timing. Specifically, we find that intertrade times are more strongly correlated for NASDAQ stocks, when data are analysed over time scales within a trading day, and that this difference is independent of the average level of trading activity of the companies (Figures 2, 3 and 4). In contrast, on time scales above a trading day there is no significant difference in the long-range correlations of companies on the two markets.

Investigating a group of companies that transferred from the NASDAQ to the NYSE, we find that intertrade times exhibit significantly stronger power-law correlations over scales from seconds to a trading day while the companies are on the NASDAQ (Figure 5). These findings suggest that market structure impacts trading dynamics, not only on a trade-by-trade basis, but over a broad range of time scales. In addition, our results imply that within a trading day the NASDAQ market structure may be more efficient than the NYSE market structure in absorbing rapid variations in trading activity in response to investors' demand [47]. In contrast, on scales above a trading day our results suggest a more coherent behavior of market agents in response to events on larger time scales, thus leading to stronger correlations in intertrade times for the companies on both markets.

Importantly, we also uncover a strong dependence between the scale-invariant features of intertrade times and stock price fluctuations: stocks with stronger correlations in their intertrade times also exhibit stronger correlations in their absolute price returns (Figure 6), indicating an influence of trading activity patterns on the dynamics of price formation. Furthermore, we show that within a trading day absolute price returns, like intertrade times, are more strongly correlated for stocks registered on the NASDAQ market (Figure 6a), and that higher price volatility on the NASDAQ is coupled with stronger correlations in intertrade times (Figure 6c). These findings suggest that market-mediated differences in transaction timing translate into differences in the scaling behavior of stock prices over a broad range of time scales.

Finally, our results do not support the hypothesis of a universal behavior in stock dynamics that is independent of individual company characteristics. In contrast to earlier studies reporting identical scaling exponents for stock price returns, volume and number of trades per unit of time [48]–[52], our findings show a strong dependance of the scaling behavior of intertrade times on the market capitalization and the average frequency of trading of individual companies (Figure 2 and Figure 3), as well as on the market structure where the companies are traded. Recent studies [32], [53] have also demonstrated that stock price returns and volume do not exhibit universal behavior, but rather depend on market capitalization. Our results show that this universality does not hold also because trading dynamics are strongly influenced by market-specific trading operations and market structure. Our results may have implications for the use of transaction timing patterns in the prediction of prices and risk management on different stock markets. These observations are of interest in the context of the continuing process of optimizing market structure to maintain the efficiency and competitiveness of U.S. stock markets [1].

## References

[1] Solomon D, Kelly K (2003) Wide SEC review may revamp structure of U.S. stock markets. Wall St Journal. Available: http://wwwwsjcom. 16 Oct 2003.

[2] Bogle JC (2003) Specialistman. Wall St Journal. Available: http://wwwwsjcom. 19 Sep 2003.

[3] US Securities and Exchange Commission (2004) SEC announces agenda for public hearing on proposed regulation national market system. US Securities and Exchange Commission Press Release. Available: http://wwwsecgov/news/press/2004-52htm. 15 Apr 2004.

[4] Bennett P, Wei L (2003) Market structure, fragmentation and market quality. Working Paper (New York Stock Exchange) 2003–04.

[5] Masulis RW, Shivakumar L (2002) Does market structure affect the immediacy of stock price responses to news? Journal of Financial and Quantitative Analysis 37: 617–648.

[6] Hasbrouck J, Sofianos G (1993) The trades of market makers: an empirical analysis of Nyse specialists. Journal of Finance 48: 1565–1593.

[7] Smith JW, Selway JP, McCormick T (1998) The Nasdaq stock market: historical background and current operation. NASD Working Paper 98–01.

[8] Peterson S (2001) Nasdaq comments on sec report on execution quality. NASDAQ Press Release. Available: http://wwwnasdaqnewscom. 8 Jan 2001.

[9] Bessembinder H, Kaufman H (1997) A comparison of trade execution cost for Nyse and Nasdaq-listed stocks. Journal of Financial and Quantitative Analysis 32: 287–311.

[10] Weaver DG (2002) Intraday volatility on the Nyse and Nasdaq. Working Paper (New York Stock Exchange) 2002–03.

[11] Christie W, Schultz P (1994) Why do Nasdaq market makers avoid odd-eighth quotes? Journal of Finance 49: 1813–1840.

[12] Gallant AR, Rossi PE, Tauchen GE (1992) Stock prices and volume. The Review of Financial Studies 5: 199–242.

[13] Lillo F, Farmer JD, Mantegna RN (2003) Master curve for price-impact function. Nature 421: 129–130. 12520292 10.1038/421129a

[14] Hechinger J (2003) Fidelity urges Nyse to revamp trading operation. Wall St Journal. Available: http://wwwwsjcom. 17 Oct 2003.

[15] Hasbrouck J (1995) One security, many markets: determining the contributions to price discovery. Journal of Finance 50: 1175–1199.

[16] Engle R, Russell J (1998) Autoregressive conditional duration: a new model for irregularly spaced transaction data. Econometrica 66: 1127–1162.

[17] Raberto M, Scalas E, Mainardi F (2002) Waiting times and returns in high-frequency financial data: an empirical study. Physica A 314: 749–755.

[18] Hausman J, Lo A, Mackinlay C (1992) An ordered probit analysis of transaction stock prices. Journal of Financial Economics 31: 319–379.

[19] Dufour A, Engle R (2000) Time and the price impact of a trade. Journal of Finance 55: 2467–2498.

[20] Jiang Z, Chen W, Zhou W (2008) Scaling in the distribution of intertrade durations of Chinese stocks. Physica A: Statistical Mechanics and its Applications 387: 5818–5825.

[21] Jiang Z, Chen W, Zhou W (2009) Detrended fluctuation analysis of intertrade durations. Physica A: Statistical Mechanics and its Applications 388: 433–440.

[22] Ruan Y, Zhou W (2011) Long-term correlations and multifractal nature in the intertrade durations of a liquid chinese stock and its warrant. Physica A: Statistical Mechanics and its Applications 390: 1646–1654.

[23] Jasiak J (1999) Persistence in intertrade durations. Working paper (York University).

[24] Ivanov P Ch, Yuen A, Podobnik B, Lee Y (2004) Common scaling patterns in intertrade times of us stocks. Physical Review E 69: 056107. 10.1103/PhysRevE.69.056107 15244883

[25] Peng CK, Buldyrev SV, Havlin S, Simons M, Stanley HE, et al (1994) Mosaic organization of dna nucleotides. Phys Rev E 49: 1685–1689. 10.1103/physreve.49.1685 9961383

[26] Taqqu MS, Teverovsky V, Willinger W (1995) Estimators for long-range dependence: an empirical study. Fractals 3: 785–798.

[27] Hu K, Ivanov P Ch, Chen Z, Carpena P, Stanley HE (2001) Effect of trends on detrended fluctuation analysis. Phys Rev E 64: 011114. 10.1103/PhysRevE.64.011114 11461232

[28] Kantelhardt JW, Koscielny-Bunde E, Rego HHA, Havlin S, Bunde A (2001) Detecting long-range correlations with detrended fluctuation analysis. Physica A 295: 441–454.

[29] Ma Q, Bartsch R, Bernaola-Galván P, Yoneyama M, Ivanov P Ch (2010) Effect of extreme data loss on long-range correlated and anticorrelated signals quantified by detrended fluctuation analysis. Physical Review E 81: 031101. 10.1103/PhysRevE.81.031101 PMC3534784 20365691

[30] Chen Z, Hu K, Carpena P, Bernaola-Galvan P, Stanley H, et al (2005) Effect of nonlinear filters on detrended fluctuation analysis. Physical Review E 71: 011104. 10.1103/PhysRevE.71.011104 15697577

[31] Yuen A, Ivanov P Ch (2005) Impact of stock market structure on intertrade time and price dynamics. Arxiv preprint physics/0508203. 10.1371/journal.pone.0092885 PMC3974723 24699376

[32] Eisler Z, Kertesz J (2006) Size matters: some stylized facts of the stock market revisited. The European Physical Journal B-Condensed Matter and Complex Systems 51: 145–154.

[33] Liu Y, Gopikrishnan P, Cizeau P, Meyer M, Peng CK, et al (1999) Statistical properties of the volatility of price fluctuations. Phys Rev E 60: 1390–1400. 10.1103/physreve.60.1390 11969899

[34] Jones C, Kaul G, Lipson M (1994) Transactions, volume and volatility. Review of Financial Studies 7: 631–651.

[35] Dayri K, Bacry E, Muzy J (2011) Econophysics of Order-driven Markets, Springer, chapter The nature of price returns during periods of high market activity. 155–172.

[36] Perelló J, Masoliver J, Kasprzak A, Kutner R (2008) Model for interevent times with long tails and multifractality in human communications: An application to financial trading. Physical Review E 78: 036108. 10.1103/PhysRevE.78.036108 18851106

[37] Gontis V, Kaulakys B, Ruseckas J (2008) Trading activity as driven poisson process: comparison with empirical data. Physica A: Statistical Mechanics and its Applications 387: 3891–3896.

[38] Politi M, Scalas E (2008) Fitting the empirical distribution of intertrade durations. Physica A: Statistical Mechanics and its Applications 387: 2025–2034.

[39] Ghysels E, Gouriéroux C, Jasiak J (1995) Market time and asset price movements theory and estimation. Working Paper 95s-32, Centre for Interuniversity Research and Analysis on Organizations.

[40] Mandelbrot BB, Fisher A, Calvet L (1997) A multifractal model of asset returns. Cowles Foundation Discussion Paper 1164, Yale University.

[41] Masoliver J, Montero M, Weiss GH (2003) Continuous-time random-walk model for financial distributions. Phys Rev E 67: 021112. 10.1103/PhysRevE.67.021112 12636658

[42] Sabatelli L, Keating S, Dudley J, Richmond P (2002) Waiting time distributions in financial markets. Eur Phys J B 27: 273–275.

[43] Mainardi F, Raberto M, Gorenflo R, Scalas E (2000) Fractional calculus and continuous-time finance ii: the waiting-time distribution. Physica A 287: 468–481.

[44] Romero N, Ma Q, Liebovitch L, Brown C, Ivanov P Ch (2010) Correlated walks down the babylonian markets. EPL (Europhysics Letters) 90: 18004.

[45] Barabási A (2005) The origin of bursts and heavy tails in human dynamics. Nature 435 207–211: barabasi2005origin. 10.1038/nature03459 15889093

[46] Vázquez A, Oliveira J, Dezsö Z, Goh K, Kondor I, et al (2006) Modeling bursts and heavy tails in human dynamics. Physical Review E 73: 036127. 10.1103/PhysRevE.73.036127 16605618

[47] Bouchaud J, Farmer J, Lillo F (2009) Handbook of financial markets: dynamics and evolution, North-Holland: San Diego, chapter How markets slowly digest changes in supply and demand. 57–160.

[48] Gopikrishnan P, Plerou V, Gabaix X, Stanley HE (2000) Statistical properties of share volume traded in financial markets. Phys Rev E 62: 4493–4496. 10.1103/physreve.62.r4493 11089066

[49] Plerou V, Stanley HE (2008) Stock return distributions: Tests of scaling and universality from three distinct stock markets. Physical Review E 77: 037101. 10.1103/PhysRevE.77.037101 18517560

[50] Bonanno G, Lillo F, Mantegna R (2000) Dynamics of the number of trades of financial securities. Physica A 280: 136–141.

[51] Gopikrishnan P, Plerou V, Amaral LAN, Meyer M, Stanley HE (1999) Scaling of the distribution of fluctuations of financial market indices. Physical Review E 60: 5305. 10.1103/physreve.60.5305 11970400

[52] Plerou V, Gopikrishnan P, Amaral LAN, Gabaix X, Stanley HE (2000) Economic fluctuations and anomalous diffusion. Phys Rev E 62: 3023–3026. 10.1103/physreve.62.r3023 11088869

[53] Eisler Z, Kertesz J, Yook S, Barabási A (2005) Multiscaling and non-universality in fluctuations of driven complex systems. EPL (Europhysics Letters) 69: 664.

